# Gluten Functionality Modification: The Effect of Enzymes and Ultrasound on the Structure of the Gliadin–Glutenin Complex and Gelling Properties

**DOI:** 10.3390/molecules30143036

**Published:** 2025-07-19

**Authors:** Daiva Zadeike, Renata Zvirdauskiene, Loreta Basinskiene

**Affiliations:** Department of Food Science and Technology, Kaunas University of Technology, 44249 Kaunas, Lithuania; renata.zvirdauskiene@ktu.lt (R.Z.); loreta.basinskiene@ktu.lt (L.B.)

**Keywords:** gluten protein, ultrasonication, modification, gluten–pea protein gels, texture properties

## Abstract

The broader application of gluten in both the food and non-food industries is limited by its lack of functional properties, such as solubility, foaming ability, and rheological characteristics. This study aimed to evaluate the physicochemical properties of proteins in various gluten products and to investigate the effects of enzymatic hydrolysis and ultrasound (US) treatment on wheat flour gluten yield, gliadin–glutenin complex structure, and gelation properties. The gelation properties of wheat gluten (GL)/pea protein (PP) treated with US and transglutaminase (TG) were studied. The results demonstrated that the ratio of low- to high-molecular-weight components in gliadins and glutenins significantly influenced the quality of commercial gluten products. A 90 min treatment of wheat flour with 24 TGU/100 g increased the yield of high-quality gluten by 32% while reducing the gliadin content by up to 6-fold. Additionally, a 30 min US treatment of 18–20% pure gluten suspensions yielded a sufficiently strong gel. The addition of PP isolate (80% protein) improved the texture of gluten gels, with the best results observed at a GL:PP ratio of 1:2. The application of TG increased the hardness, consistency, and viscosity of GL-PP gels by an average of 5.7 times while reducing stickiness. The combined TG and US treatments, along with the addition of PP, notably increased the levels of lysine, isoleucine, and tryptophan, thereby enhancing both the nutritional quality and amino acid balance of the final product.

## 1. Introduction

Wheat is one of the most important cereal grains globally, serving as a staple food and a key source of nutrients for a large portion of the world’s population. Its unique viscoelastic properties, primarily attributed to gluten, make wheat flour indispensable in baking and various food applications. However, the functional limitations of gluten, such as insufficient solubility or weak gelling capacity in certain formulations, restrict its broader industrial use beyond conventional bakery products.

Gluten, composed of gliadin and glutenin, is primarily responsible for the unique viscoelastic properties of wheat-based dough. While the demand for wheat gluten continues to grow, driven by its expanding use across both food and non-food industries, its broader application is hindered by the lack of certain key functional properties, such as solubility, foaming capacity, and emulsifying ability [1]. Although the gelling properties of plant protein isolates are critical in a range of food products, including meat analogs, tofu, and dairy alternatives, the gel-forming ability of these proteins, including gluten, remains relatively weak [2,3]. Consequently, enhancing the functional characteristics of gluten and other plant proteins has become both a major challenge and a crucial objective for food scientists and technologists [4].

Food manufacturers are looking for innovative technologies for both products and processes that would allow for a wider range of protein applications while maintaining product quality and competitiveness. One such innovative additive is enzymes, which are considered the best alternative to chemical compounds because they are generally recognized as safe (GRAS) and do not remain active after cooking. Enzymes act through different mechanisms and can induce changes in the shape of the polymerized subunits of proteins and increase their solubility, thereby enhancing their functional properties.

For efficient gluten separation, xylanases (endo-1,4-β-xylanases, EC 3.2.1.8) are commonly used in the industry to cleave the linear xylan chain of arabinoxylans (AX), thereby reducing their degree of polymerization and increasing the efficiency of protein extraction [5]. In recent years, microbial transglutaminase (TG) has been widely used to improve the gelling and textural properties of food proteins, including soy, wheat, pea, whey, casein, and myofibrillar proteins [6,7]. Since vegetable proteins contain sufficient lysine but a relatively low amount of glutamine, and gluten contains a high amount of glutamine but less lysine, the combination of vegetable proteins and gluten, catalyzed by TG, can promote the formation of additional covalent bonds and thus enhance gelation properties. The development of ultrasound (US) technology in the food industry has recently attracted much attention worldwide. Various methods, including physical [8], chemical [9], and enzymatic [10,11], have been widely used, but little is known about the effect of US technology on the functional properties of cereal proteins, especially wheat gluten proteins. In the past decade, US technology has been increasingly applied to improve various biotechnological processes in food systems, owing to its ability to induce rapid structural changes without the need for additional additives. High-intensity US (frequency 16–100 kHz, power 10–100 W/cm^2^) generally is used to break down polymers or intensify chemical reactions [12]. In protein systems, sonication has been shown to improve functional properties such as solubility, emulsification, foaming, and gel formation [13], with the extent of these changes being strongly dependent on the applied US power and processing duration [14,15]. These improvements are often attributed to the partial disruption of hydrophobic interactions and the unfolding of protein structures, leading to increased exposure of reactive or functional groups [16,17]. Although the individual effects of US and enzymes like TG on protein structure have been investigated, their combined use remains largely unexplored in cereal proteins. TG catalyzes covalent cross-links between glutamine and lysine residues, enhancing protein network strength and stability. However, its activity may be limited by the inaccessibility of reactive sites within tightly packed protein matrices. US pretreatment has the potential to overcome this limitation by inducing partial protein unfolding and increasing the availability of enzymatic binding sites. Therefore, the synergistic application of US and TG could serve as a novel and promising strategy for modifying wheat gluten structure and functionality more effectively than individual treatments.

This work aims to evaluate the physicochemical properties of different commercial gluten proteins and investigate the effect of transglutaminase and hemicellulases on gluten extraction yield, as well as on the structure of the gliadin–glutenin complex and the gel-forming capacity of gluten–pea protein blends.

## 2. Results and Discussion

### 2.1. Characterization of the Commercial Gluten and Gluten-Forming Protein Fractions

The main quality indicators of the tested commercial gluten samples, presented in Table 1, confirms that the GI, one of the main criteria of the quality of gluten, depends on the protein content of the gluten (*r* = 0.838) and strongly affects the water absorption properties of the gluten protein (*r* = 0.999).

**Figure 1:**
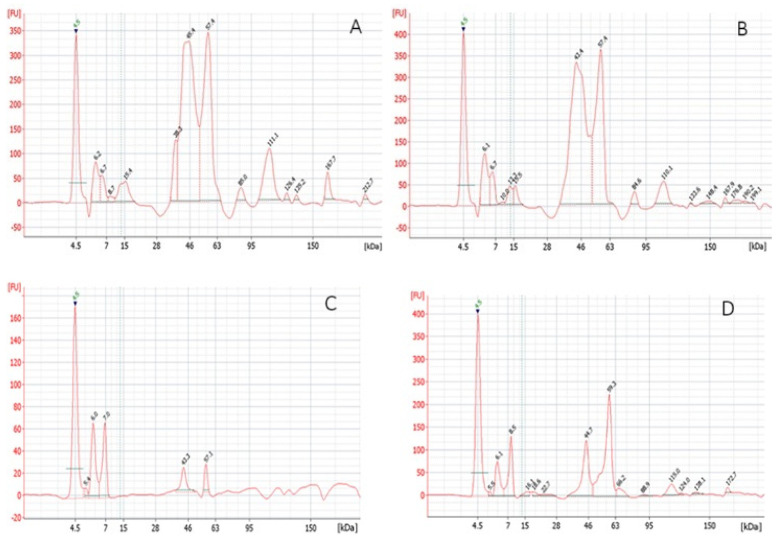
The electrophoregrams of gliadins (**A**,**B**) and glutenins (**C**,**D**) of L096F (GI94) (**A**,**C**) and F8C01 (GI71) (**B**,**D**) gluten samples.

The quantitative analysis of gluten proteins is presented in Table 2, and the results of qualitative analysis of gluten protein fractions are presented in Figure 1. According to the analysis, gluten GI71 stands out with the lowest gliadin content (50.3 g/100 g protein) and with a glutenin-to-gliadin ratio (Gla/Glu) of 1.01, compared to the G94 gluten sample (65.21 g gliadin/100 g protein, and Gla/Glu = 1.87). In the analysis of different commercial gluten samples, the Gla/Glu ratio was negatively correlated with the gluten index (*r* = −0.998), protein content (*r* = −0.812), and glutenins (*r* = −0.998) and positively correlated with gliadins (*r* = 0.998).

According to Schopf et al., in vital wheat gluten, the gliadin-to-glutenin ratio varies between 1.5 and 2.7 [18]. The study by Dhaka and Khatkar has also shown the gliadin-to-glutenin ratio ranging from 0.75 to 1.16, whereas the high-molecular-weight glutenin (HMW-GLN) subunits to low-molecular-weight glutenin (LMW-GLN) subunits ratio of these varieties ranged from 0.31 to 0.93 [19].

The quantitative differences between HMW and LMW gliadin and glutenin were analyzed by comparing protein electrophoresis patterns (Figure 1). In the gluten GI94 and GI71 samples, the high-molecular-weight gliadin (HMW-GLD) components (84.5–212.7 kDa) were identified, while the most intensive peaks (330–380 FU) were identified for the LMW-GLD subunits (42.4 to 57.4 kDa) (Figure 1A,B). These components constituted most of the gliadins (~75%). In the gluten GI94, the HMW-GLD subunit peaks were of higher intensity (17–115 FU) (Figure 1A), compared to the gluten GI71 (10–60 FU) (Figure 1B) with the almost same peak intensity of the LMW subunits.

Based on the results, it can be stated that the HMW-GLD significantly affected gluten quality. The higher GI value of sample GI94 can be due to the higher ratio of HMW- to-LMW-GLD (0.05–0.34) compared to sample GI71 (0.03–0.16) (Figure 1A,B).

The glutenin subunits of 43–66 kDa were detected in both gluten samples (Figure 1C,D), while more intensive peaks (20–220 FU) of those proteins were detected in gluten GI94 (Figure 1C), and glutenin subunits of 16–22 kDa and proteins with an MW between 90 and 172 kDa were detected additionally in the gluten GI71 (Figure 1D), indicating lower quality. Also, the HMW to LMW-GLN subunits ratio of 0.11 and 0.48, respectively, in these gluten samples confirmed the higher quality of gluten GI94 compared to GI71.

According to the literature, glutenins are polymeric proteins consisting of HMW subunits of 75–120 kDa and LMW subunits of 20–55 kDa [20]. Gliadins are responsible for the viscosity of the gluten network, while glutenins are responsible for elasticity and strength [21]. Although HMW-glutenins account for nearly 10% of gluten proteins, these subunits impact 50–70% of the technological quality of the wheat grain, while LMW-glutenin subunits account for about 50% of gluten proteins and impact 30% of wheat technological quality [22].

It was assumed that the higher amount of LMW-glutenins and gliadins form a more stable network, affecting gluten elasticity, i.e., results in higher GI values [23]. Both the quantity of HMW-glutenin subunits and the subunit composition influence gluten viscoelasticity by modifying the polymer size distribution and aggregative properties of gluten proteins [24].

According to the results, the quality of the tested commercial gluten samples was significantly influenced by the gliadin fraction, as well as the ratio of HMW to LMW of both gliadin and glutenin, which higher values increase gluten resistance and extension. As glutenin provides strength and elasticity, while gliadin contributes to viscosity and extensibility, a balanced ratio significantly impacts the gelling properties of the resulting dough and is crucial for innovative gluten applications, such as the production of renewable bio-packaging materials [25].

### 2.2. The Application of Transglutaminase and Hemicellulases for Wheat Gluten Yield and Quality Modification

**Figure 2:**
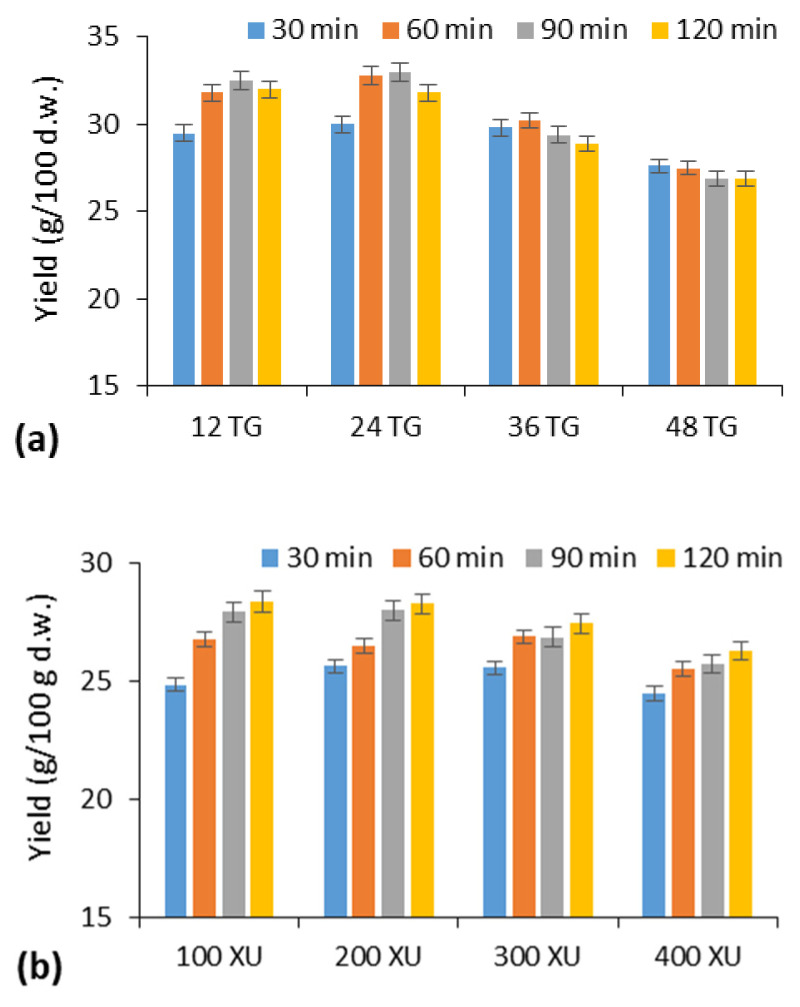
The effect of transglutaminase (TG) (**a**) and hemicellulases (Sherazyme Plus) (**b**) on wheat flour (GI92) gluten yield.

The application of transglutaminase (TG) for increasing gluten yield and improving gluten quality was analyzed through a comparison with Shearzyme hemicellulases (SH). The results showed (Figure 2a) that the influence of TG on gluten yield depended on the process duration and enzyme amount. The highest yield was obtained after 90 min when wheat flour was treated with 24 TGU/100 g. In this case, 32% more wet gluten was isolated compared to the control sample (24.32 g/100 g flour) (Figure 2). In addition, the use of TG allowed increasing the GI values of wheat gluten from 92 (initial value) up to 99–100% (Figure S1). However, higher TG activity (36–48 TGU/100 g) already reduced gluten yield by an average of 8.5–14% (Figure 2), as well as GI values up to 89.6–96.5%, with longer duration time (90–120 min) (Figure S1).

The highest gluten yield (29.42 g/100 g) was obtained after 90 min of hydrolysis using an amount of SH corresponding to 200 XU/100 g flour (Figure 2b). However, in this case, the gluten yield increased by 19% compared to the control sample, but the gluten obtained had a lower GI (96.8%) than when TG was used. Higher enzyme activities of SH and longer treatment time reduced both the gluten yield and the GI values (Figure S1).

According to the obtained results, TG enables more efficient gluten formation compared to hemicellulases. Although the latter enzymes are suitable for gluten isolation, the required processing time of 90 min is too long, as it negatively affects gluten quality. The optimal amount of TG for efficient gluten formation is 24 AU/100 g, with a 60 min treatment, achieving a GI of 100%.

According to the literature, transglutaminase (γ-glutamyltransferase, EC 2.3.2.13) catalyzes the acyl transfer reaction between glutamine and lysine residues, between which covalent intermolecular or intramolecular bonds are formed, which changes the quality of gluten and has a positive effect on its separation [26]. The action of the enzyme transglutaminase is based on the modification of wheat proteins, mainly HMW glutenin subunits, by creating new bonds between protein chains, enhancing protein network formation [27], while excessive TG activity negatively affects gluten quality and, consequently, technological properties, forming a weak gluten network and reducing product quality [28].

### 2.3. The Influence of Enzymatic Treatment on Quantitative and Qualitative Changes in Wheat Flour Gluten Proteins

The gluten was extracted from wheat flour treated with TG and SH enzymes. Gliadin electropherograms (Figure S2) revealed that both TG and SH resulted in higher gliadin quantities compared to the control sample, as indicated by more intensive peaks in the electropherograms. In both cases, enzymatic hydrolysis increased the peak intensities from 25–112 FU to 380–430 FU of LMW-GLD subunits (44.6–87.9 kDa) and HMW-GLD subunits (110.6–240 kDa), respectively. In all analyzed samples, after 90 min. of enzymatic hydrolysis, the majority of gliadins consisted of 44–57 kDa subunits. Under the action of TG, higher peak intensities for LMW-GLN subunits were identified (up to 40 FU), compared to the action of the SH enzymes (up to 20 FU) (Figure S3). Moreover, the high intensity peaks of 44.3–59.2 kDa of gliadin subunits indicate the decomposition of HMW gliadins. Gluten modification by TG involves the enzyme deamidating glutamine residues in gluten proteins, which can be a crucial step in gluten digestion. LMW glutenin subunits, along with gliadins, are susceptible to degradation by TG, and some studies suggest that the remaining decomposed gliadins might be associated with LMW glutenins [29].

In terms of glutenin and gliadin subunits distribution, the treatment with 24 AU of TG significantly increased (*p* < 0.05) the glutenin yield, with processing time increasing from 30 to 90 min. In contrast, the effect of hemicellulases was less pronounced and more variable (Table 2). The highest glutenin yield (63.8 g/100 g d.w.) and the lowest gliadin-to-glutenin ratio (Gla/Glu = 0.57) were observed after 90 min of TG treatment, while under SH treatment, a higher Gla/Glu ratio (0.99) was obtained compared to the control. After 30 and 60 min of wheat flour treatment with SH enzymes, the Gla/Glu ratios were 0.86 and 0.89, respectively, indicating that the extracted gliadin fraction was significantly higher (*p* < 0.05) than in samples treated with TG for the same durations.

Rosell et al. [30] stated that the allergenic properties of gluten can be reduced through processing with transglutaminase (TG); therefore, TG may potentially be used in the production of low-gluten wheat products suitable for consumers with gluten intolerance [31].

According to the literature, in the molecular weight range between 35 and 58 kDa, the wheat toxic α- and ω-gliadins are determined [32]. Moreover, our study also confirmed that during wheat flour hydrolysis with TG and SH, these LMW-gliadin components were not degraded, even with prolonged processing. In hydrolyzed wheat flour samples, α-gliadins of 44–47 kDa were detected, which are characterized as more toxic than γ-gliadins (57 kDa).

### 2.4. The Effect of Ultrasound on the Gelling Properties of Gluten

The results of the influence of US treatment and protein concentration on the formation of gluten protein gels are presented in Table 3. The gluten gel-forming capacity depended on the duration of US treatment and the protein concentration. With a 10 min US treatment, gel formation occurred in both gluten samples when the protein concentration was generally within the range of 18–20%. By increasing the US exposure time to 20 and 30 min, gel formation occurred at 14–20% protein concentration in the suspension (Table 3).

When comparing low- and high-GI gluten gels, it was found that with GI94 gluten, gelation occurs after 10 min of US treatment at a protein concentration of 14%. Strong gels were formed when suspensions with 18 and 20% protein were sonicated for 20–30 min. In contrast, the formation of GI71 gluten gels consistently requires a higher protein concentration (18–20%) and a longer US treatment duration (30 min) to achieve strong gelation. It is worth noting that after 30 min of US treatment, no significant differences in gel formation were observed among the different gluten samples (Table 4). In summary, the optimal conditions for proper gluten gel structure formation, regardless of gluten quality, were identified as 18% protein concentration and 30 min US treatment.

Relying on inter- and intra-interactions of glutenin and gliadin proteins, wheat gluten could form a net with air-holding. The interaction of unfolded proteins might produce an improvement of the foam stability of ultrasound-treated gluten. In the study of Jambrak et al. [33], the ultrasonication significantly changed the texture of model systems prepared with soy protein concentrates that gelled during US treatment with a 40 kHz bath for 15 min at 45 °C. In the research of Zhang et al. [14], the condition of 20 kHz of ultrasound treatment for 10 min with continuous flow did not cause major changes in the protein electrophoretic patterns of gluten samples. This condition may somehow cause denaturation that could expose hydrophobic regions of wheat gluten [34].

### 2.5. The Effect of Ultrasound on the Formation of Gluten and Pea Protein Gels

Since legume protein isolates have a high protein content, they are widely used in the food industry for the development of new food products. Pea protein (PP) isolate is valued in the food industry for its high lysine content and nutritional value. Although it forms a weaker and less elastic gel than soy protein, it causes fewer allergic reactions than gluten or soy proteins [35].

**Figure 3:**
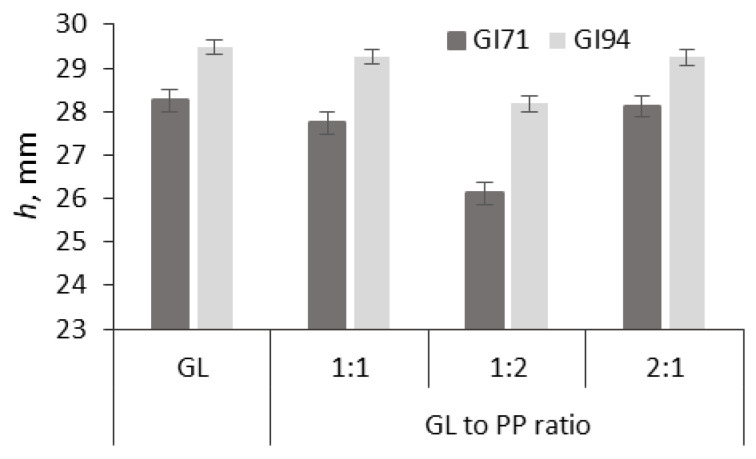
The effect of ultrasound on *h* parameter (measured after 150 s of processing) of different gluten (GL) and its blends with pea protein (GL-PP) at different ratios. GL—18% concentration gluten gel; GI71, GI94—commercial gluten samples of different gluten index (GI) values.

The effect of PP addition on the formation and texture of US-assisted gluten protein gels was investigated (Figure 3). Suspensions of different gluten (GL) samples (GI71 and GI94) and PP blends at a ratio of 1:1; 2:1; 1:2, respectively, with a total protein content of 18% were analyzed.

The results of the texture analysis of GL-PP gels showed that in all cases, the addition of PP reduced the *h* parameter values, i.e., it increased the viscosity of the mass compared to the control samples. The lowest *h* value, and thus the highest viscosity, was observed at a gluten-to-PP ratio of 1:2. For the GI94-PP sample, the *h* value of the gel decreased by 4.8% compared to the GI94 sample (*h* = 29.5 mm). After US treatment of the GI71-PP blend, the *h* value decreased by 7.6% for this gel compared to the GI94 sample (*h* = 28.17 mm). It can be assumed that the addition of PP had a more positive effect on the texture of gluten gels with a lower gluten GI. Among the gluten-PP gels, the sample with a 1:2 component ratio formed the strongest and most homogeneous structure.

The observed reduction in the *h* parameter, indicating increased viscosity, suggests improved internal cohesion within the GL-PP gel matrix. This increase in viscosity is typically associated with enhanced resistance to flow and deformation, which, in practical terms, contributes to a firmer, more stable gel. Additionally, the formation of a more homogeneous structure at a GL:PP ratio of 1:2 implies uniform protein distribution and stronger intermolecular interactions. These structural improvements can translate into better texture in terms of sensory perception, such as smoother mouthfeel, reduced graininess, and greater elasticity, qualities desirable in various food applications like meat analogs or high-protein baked goods. Therefore, the rheological changes observed in this study are closely aligned with textural enhancements relevant to both product quality and consumer acceptance.

The obtained results are consistent with the literature data, indicating that protein gels composed of PP isolate and gluten exhibit higher viscosity, elasticity, and strength [36]. According to Munialo et al. [37], under heating, pea proteins are hydrolyzed into peptides, 50% of which assembled into fibrils. The formation of branched fibrillar structures led to the development of HMW protein aggregates. The authors noted that gel formation was most effective when the pH of the protein suspension was shifted from 7 to 5, demonstrating that PP fibrils can be effectively used for protein gel production. Of course, temperature, the degree of protein denaturation, and the protein concentration are particularly important for gel formation, as they determine gel strength [38]. Thus, it can be concluded that the PP isolate can be used for the improving the texture and stability of the gluten gels. By appropriately selecting the ratio of PP to gluten as 1:2 in this experiment, it is possible to significantly improve gel properties and adapt them effectively for use in the food industry.

### 2.6. Application of Transglutaminase to Improve Gluten and Pea Protein Gel Quality

According to the literature, TG improves protein solubility, and during gluten gelation, high-molecular-weight polypeptides can be formed [39]. In this way, rheological properties of vegetable protein gels are improved, forming a denser, more homogeneous gel structure [40].

**Figure 4:**
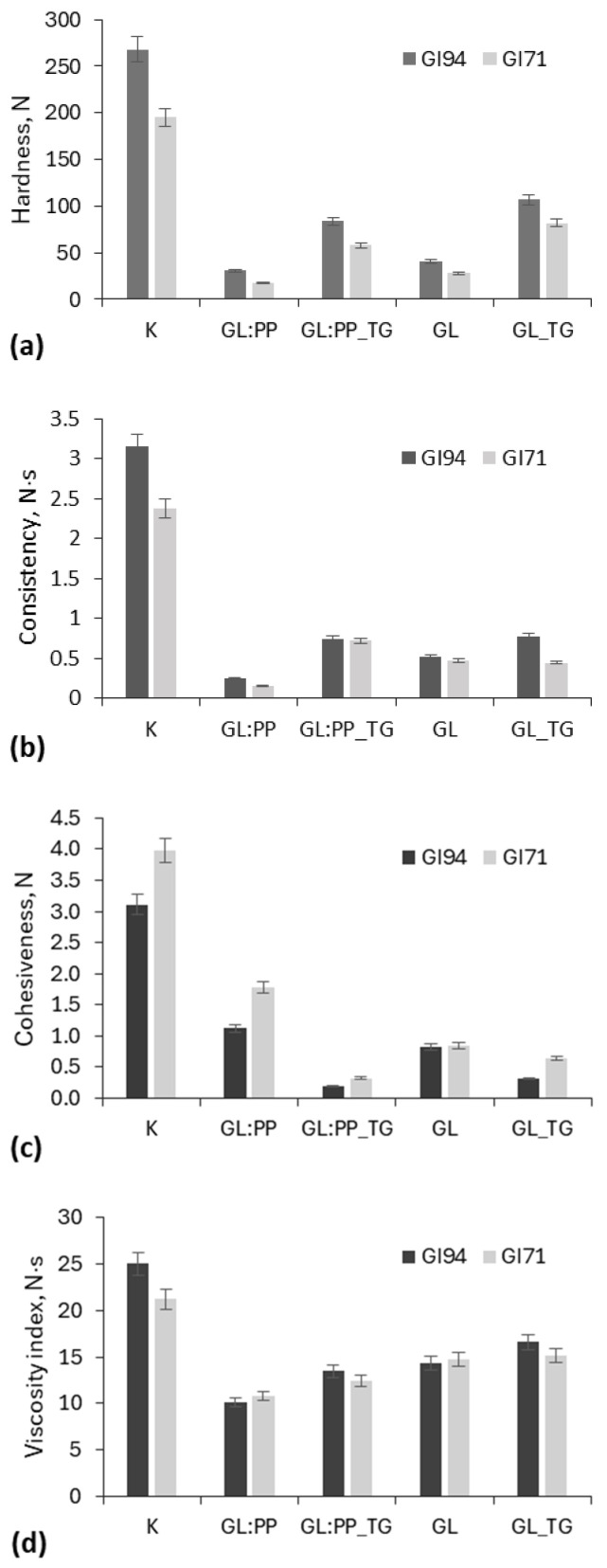
The effect of transglutaminase (TG) on hardness (**a**), consistency (**b**), cohesiveness (**c**), and viscosity (**d**) of different gluten (GL) and pea protein (PP) (1:2) gels obtained after ultrasonication (protein concentration in suspension 18%). K—gluten gels obtained by heating protein suspension at 100 °C.

The texture of GL-PP gels was evaluated based on their strength, viscosity, elasticity, homogeneity, and stability (Figure 4). The obtained results showed that, in all cases, the GI94 gluten gels exhibited greater hardness and consistency compared to the GI71 gluten gels.

The highest hardness and consistency values were observed in the gluten gels obtained by heating the protein suspension at 100 °C (Figure 4a,b). Among the by-US-at-60 °C-treated samples, gels prepared from GI94 gluten exhibited higher hardness and consistency (by 28 and 24%, respectively) compared to the GI74 gluten gels. The addition of PP reduced the hardness and consistency of the GL-PP gels by an average of 29 and 59%, respectively, compared to the corresponding pure gluten gels.

Moreover, the gels obtained after US treatment showed significantly (*p* < 0.05) lower stickiness and viscosity compared to those heated at 100 °C (control sample) (Figure 4c,d). The PP increased the stickiness and decreased the viscosity of gluten gels by 43% and 28%, respectively, depending on the type of gluten used. In all cases, the use of TG increased the hardness, consistency, and viscosity of the gels while reducing their stickiness compared to the gels without TG.

According to the literature, ultrasonication at three power levels (100, 200, and 300 W) for 10 min significantly increased solubility, oil holding capacity, emulsifying activity, and foam capacity of pea protein [41]. Riquelme et al. reported forming weak gels with pea and lupin proteins treated after 5 and 10 min of US, respectively, whereas rice protein did not form gels [42]. Furthermore, US treatment time resulted in more structured gels after longer treatment times (US 15 min). These findings and our study suggest that US treatment enhances the gelling properties of pea and lupin proteins, making them more suitable for plant-based food applications such as yogurts or desserts.

Visual evaluation of the texture of the obtained gels revealed that gluten gels heated at 100 °C formed a firm but non-homogeneous structure (Figure S4). Gluten and PP blend suspensions treated with US formed firm and homogeneous gels, while the use of TG reduced the surface stickiness and moisture of the gels. Gels made from GI94 gluten were characterized by a gray-tone color, whereas the surface of gels from GI71 gluten exhibited a porous structure.

**Figure 5:**
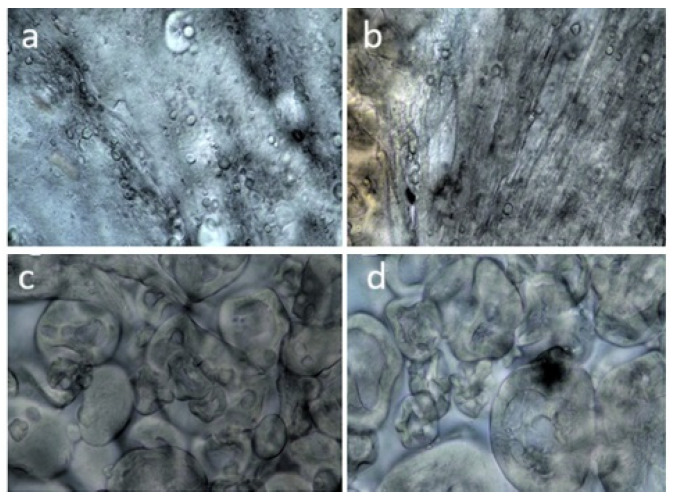
Microscopic analysis of gels, formed after ultrasound pretreatment of 18% gluten–pea protein suspensions. Gels: gluten (**a**); gluten treated with transglutaminase (TG) (**b**); gluten–pea protein (**c**); gluten–pea protein treated with TG (**d**).

Microscopic analysis of the gels (Figure 5a) showed that the structure of US-treated gluten gels was not uniform, with visible water molecules distributed throughout the structure. When gluten was treated with US and subsequently processed with TG, a more uniform gel structure was formed, characterized by the appearance of protein fibers with small amounts of water molecules between them (Figure 5b). In the microscopic image of the GL-PP gels, pea protein fibrils were observed dispersed within the gluten matrix (Figure 5c). Under the influence of TG, a denser network of PP protein fibrils formed, which reinforced the gel structure (Figure 5d). Protein treatment with US at 60 °C promoted the formation of a stronger gel structure, facilitated by the formation of insoluble protein aggregates between gluten and PP molecules, resulting in a homogeneous and cohesive structure upon TG treatment.

According to Jambrak et al. [33], treating whey protein isolate and whey protein concentrate with 20 kHz ultrasound for 15 to 30 min reduced the size of protein particles and increased their surface area. The authors also reported that exposing these proteins to 40 kHz ultrasound altered the molecular weight of the protein fractions.

Studies by the other authors [43] showed that in gels of similar composition, two distinct phases of soy protein isolate (SPI) and gluten could be observed using confocal laser scanning microscopy. It is believed that these phases are relatively pure and composed predominantly of either soy protein isolate or gluten [44]. To properly understand the water-holding capacity in different gels, it is important to consider how water is distributed between SPI and gluten before gel formation. Gluten hydration is limited due to the formation of cross-links during hydration. Experimental data on the water distribution between SPI and gluten, depending on their ratio, revealed that SPI binds more water than gluten [45].

### 2.7. The Qualitative Changes in Proteins During Gel Formation

Chromatographic analysis revealed that the combined treatment of GL and PP blends with US increased the intensities of LMW protein peaks (between 47 and 56 kDa) in the prolamin fraction of the gels (Figure S5). In the control gels, prepared by heating the protein suspension at 100 °C, the low-molecular-weight protein peaks exhibited lower intensities (between 80 and 240 FU) compared to those treated with US (150–420 FU). The increase in LMW protein content was associated with enhanced protein solubility. No significant changes were observed between the prolamin subunits of gels prepared from the gluten GI94 and PP mixture (Figure S5). However, the intensities of protein peaks were higher in the US-treated samples (235–430 FU) compared to those hydrolyzed with TG (190–410 FU). More pronounced changes in the prolamin fraction were observed in gels from the gluten GI94 and PPI samples treated with US or TG. In the gluten GI71 and PP suspension heated at 100 °C, the intensities of the HMW protein peaks (25–50 FU) increased compared with those treated with US and TG (15–20 FU) (Figure S5).

The analysis of the effects of TG and US treatments on the amino acid profiles of GL–PP gels (Table 4) revealed that the combined treatments resulted in a 15.8% increase in the measured glutamine content, which may have facilitated the formation of a stronger protein network, as evidenced by the textural properties of the gels (Figure 4). According to Dupont et al. [46], TG promotes the formation of intra- and intermolecular covalent bonds between glutamine and lysine residues, thereby increasing the molecular weight of gluten. These findings suggest that US treatment alters the protein structure, making it more accessible for TG-induced cross-linking.

Additionally, the combined pretreatments of the GL-PP suspensions resulted in an apparent increase of approximately 16.7% and 17.3% in the measured levels of isoleucine and tryptophan, respectively, as well as a 19.2% increase in lysine (Table 4). The analysis of gluten and GL-PP gels further revealed that the combined TG and US treatments enhanced the recovery of total amino acids following acid hydrolysis, compared to US treatment alone.

The observed increase in lysine, isoleucine, and tryptophan levels can primarily be attributed to the addition of PP isolate, which is naturally rich in essential amino acids. However, it is also possible that the applied treatments, particularly US, may have contributed to improved protein unfolding and structural modifications, potentially enhancing the extractability or bioavailability of these amino acids. While the quantitative increase is likely driven by the PP content itself, the role of US and TG treatments in modifying protein conformation and exposing amino acid residues should not be excluded and warrants further investigation.

The TG treatment reduced the apparent availability of cysteine by 45–60%, compared to US. The observed decrease in measured cysteine is likely due to its participation in disulfide bond formation during the TG-induced cross-linking process, which reduces the quantity of free thiol groups available for detection. As a sulfur-containing amino acid, cysteine is critical for maintaining the stability of cellular proteins and supporting compounds such as heparin, vitamin B, and alpha-lipoic acid. It also promotes gastrointestinal health, facilitates signal transduction between the central nervous system and other cells, and acts as a detoxifying agent [47].

According to Wang et al. [48], transglutaminase (TG) promotes the formation of cross-links between gluten proteins with higher glutamine content, while the application of ultrasound in gel structuring increases the glutamine levels. Other studies have shown [49] that protein hydrolysates generated by enzymatic treatment combined with ultrasound can exert certain health-promoting effects related to the antioxidant activity of amino acids such as histidine, tryptophan, phenylalanine, and tyrosine. In agreement with the literature, protein treatments prior to enzymatic hydrolysis enhance the release and bioavailability of bioactive peptides, making them more accessible to digestive tract enzymes [49]. The treatment of soy protein isolate (SPI) and gluten with high-intensity ultrasound significantly improves the gelling properties of SPI–gluten blends by reducing particle size and strengthening hydrophobic and sulfhydryl interactions at the gel surface, thereby facilitating disulfide bond formation and hydrophobic interactions that, in the presence of TG, result in a dense and homogeneous gel network [16].

In summary, treatment with transglutaminase and ultrasound may offer a novel technology for enhancing the functional properties of gluten, thereby broadening its potential applications in the food industry.

## 3. Materials and Methods

### 3.1. Commercial Gluten and Pea Protein Isolate

Three different commercial gluten samples obtained from the Lithuanian starch producing company were analyzed. The main quality parameters of gluten products are presented in Table 5.

Pea protein isolate (moisture 5.6%, protein 80%, fiber 4.1%, fat 5.5%, ash 2.1%) (MYPROTEIN, The Hut.com Ltd., Cheshire, UK) was used for gel production.

### 3.2. Flour and Enzymes

Commercial wheat flour (moisture 14.5%, protein 13.3%, wet gluten 24.32%, GI 92%, 0.54% ash) with low gluten quality (gluten index 92%) was obtained from the mill SC ‘‘Malsena Plus’’ (Vievis, Lithuania). The enzyme transglutaminase (Fraken Biochem Co., Qingdao, China) with the activity of 100–120 TGU/g and liquid hemicellulase preparation Shearzyme^®^ Plus (Novozymes, Bagsvaerd, Denmark) with cellulase (350 EGU/g) and endo-*β*-xylanase (250 FXU-S/g) activities and a side activity of *β*-glucanase were used in this study. The working range of transglutaminase is at pH 5.0–8.0 and 40–50 °C temperatures. Hemicellulases are active at pH 4.5–6.5 and 50–75 °C temperatures.

### 3.3. Chemical Analyses

The moisture, protein, and ash contents were determined according to the AOAC Official Methods [50]. The wet gluten and gluten index (GI) was determined using a Glutomatic 2200 device (Perten instruments, Hägersten, Sweden). Each gluten sample (10 g) was analyzed in triplicate.

### 3.4. Determination of Hydration Properties

The water absorption capacity (WAC), water solubility (WS), and swelling power (SP) of gluten were determined by mixing the sample (1 ± 0.001 g) with 6 mL of distilled water, following incubation in a 30 °C water bath (Memmert GmbH, Schwabach, Germany) for 30 min. Then the samples were centrifuged (6000 rpm; 20 min), the resulting centrifugate was poured into weighed glass tubes, and wet sediment was weighed. The centrifugate was dried at 105 °C to constant weight. The WAC, WS, and SP were calculated according to Gaizauskaite et al. [51].

### 3.5. Amino Acids Analysis

For the test, a gluten sample (1 ± 0.001 g) was mixed with 7.5 mL of 6 M HCl and kept for 24 h at 110 °C. Then the sample was centrifuged (6000 rpm, 20 min); 2.5 mL of the centrifugate was mixed with 7.5 mL of distilled water and filtered through a 0.45 µm membrane filter (PTFE). Amino acids were determined by the Ultrafast Liquid Chromatography (UFLC) (Shimadzu Corporation, Kyoto, Japan), using a YMC Triart C_18_ column and a fluorescence detector (RF-20Axs). Analytical conditions: mobile phase, solvent A (20 mmol/L potassium phosphate buffer (pH 6.5) and solvent B (45/40/15; acetonitrile/methanol/water), flow rate 0.8 mL/min, column temperature 35 °C. The amino acids standards (A9781 Sigma-Aldrich, Steinheim, Germany) of 0.5 μmol/mL concentration, except for L-cystine at 0.25 μmol/mL in 0.2 N sodium citrate, pH 2.2, were analyzed. A five-level calibration set was used, covering a concentration range of 0.006–0.20 μmol/mL, except for alanine and cysteine, each covering a concentration range of 0.06–1.00 μmol/mL.

### 3.6. Enzymatic Treatment Procedure

For the experiment, a working solution of Shearzyme Plus (SH) (250 XU/mL) was prepared in 0.1 M sodium acetate buffer (pH 4.7). For the experiment, a wheat flour sample (5 ± 0.001 g) was mixed with 20 mL of sodium acetate buffer and a corresponding amount of SH solution (100–400 XU/100 g flour). Transglutaminase (TG) working solution (12 TGU/mL) was prepared in a 0.1 M phosphate buffer (pH 7.2). For wheat flour gluten modification, the amounts of 12–48 TGU/100 g flour were used. The TG and SH samples were incubated for 120 min in a 40 °C or 50 °C water bath (Memmert GmbH, Schwabach, Germany), respectively, with shaking (200 rpm). To assess the efficiency of the enzymatic treatment, the samples were taken after 30, 60, 90, and 120 min of incubation; then, enzymes were inactivated by raising the temperature to 90 °C for 10 min. After enzymatic and thermal treatments, samples were freeze-dried, and the obtained powders were collected for further analysis.

### 3.7. Gluten Protein Fractionation

Osborne fractionation of gluten protein in commercial gluten and untreated and enzymatically treated wheat flour was performed following the method as described by Teka et al. [52] with slight modifications.

For gliadin extraction, a gluten sample (25 ± 0.1 mg) was mixed with 800 µL of 70% aqueous ethanol solution, the samples were incubated for 30 min at room temperature, with vigorous shaking every 10 min. After extraction, the sample was centrifuged (2000 rpm, 5 min), the resulting liquid fraction was collected (gliadin fraction I). A total of 800 µL of 70% aqueous ethanol solution was repeatedly poured onto the sediment, extraction was performed analogously, the sample was centrifuged and the resulting centrifugate was collected (gliadin fraction II). The extraction was repeated, thus obtaining gliadin fraction III in solid residue.

For glutenin extraction, the precipitate obtained after gliadin III extraction was added with 400 µL of 50% 1-propanol + 1% DTT solution, the samples were incubated for 30 min at room temperature, with intensive shaking every 10 min. After extraction, the samples were centrifuged (2000 rpm; 5 min), the resulting centrifugate was decanted (glutenin fraction I). Centrifugate (300 µL) from glutenin fraction I was taken and mixed with 75 µL of 100% propanol and 1% DTT solutions, the mixture was incubated for 60 min in a 4 °C water bath. After, the sample was centrifuged, liquid part was decanted, and the precipitated glutenins were dissolved in 100 μL of 50% 1-propanol with 0.082 M Tris-HCl (pH = 7.5) and 2 M urea + 1% DTT solution and subjected for the protein analysis. Gliadin and glutenin protein contents were expressed as g/100 g protein.

### 3.8. Capillary Electrophoresis of Proteins

The capillary electrophoresis system an Agilent 2100 Bioanalyser (Agilent Technologies, Santa Clara, CA, USA) was used for the protein fractionation and analysis. A sample buffer consisting of 0.4 mL of 2M urea, 50% 1-propanol, 0.1 M DDT (Dichlorodiphenyltrichloroethane) and 0.082 M Tris-HCl (buffer pH 8.0) was prepared. For the analysis, the gliadin or glutenin sample (100 µL) was mixed in the test tubes with sample preparation buffer (2M urea, 15% glycerol, 0.1 M DDT and 0.1 M Tris-HCl (pH 8.0), 150 µL of buffer solution and 75 µL of distilled water were mixed by vigorous shaking. The samples were kept in a 25 °C ultrasonic bath for 15 min, then centrifuged (10,000 rpm, 5 min) in a microcentrifuge (MC-24R, Benchmark Scientific, Sayreville, NJ, USA). The 4 µL of resulting centrifugate was mixed with 2 µL of denaturing solution (200 µL of sample buffer and 7 µL of mercaptoethanol), were centrifuged and kept in a boiling water bath for 5 min. After heating, 84 µL of distilled water was added to each sample and mixed. A special microplate was filled with the gel staining solution, then the wells were filled with test samples and protein standards solution. The proteins analysis data were processed using the Agilent 2100 Expert (ver. B.02.12, Agilent Technologies, Santa Clara, CA, USA) software.

### 3.9. Ultrasound-Induced Gelation Procedure

#### 3.9.1. Sample Preparation Procedure

In the first stage of the experiment, commercial gluten (GL) suspensions were prepared in distilled water at different protein concentrations (14, 15, 16, 18, and 20%) and treated with US (37 kHz; 160 W) at 60 °C for varying durations (10, 20, and 30 min) to determine the optimal protein concentration and sonication time for strong gel formation. In the next stage, suspensions (pH 6; 18% protein) of different gluten and pea protein (PP) isolate blends were prepared at ratios of 1:1, 2:1, and 1:2, respectively, and sonicated for 30 min to identify the optimal GL/PP ratio for gel formation. In the third stage, the US-induced gels were prepared using suspensions (18% protein, pH 6) of commercial gluten or the optimal GL-PP blend, both untreated and treated with transglutaminase (TG). Before sonication, samples with TG were inactivated by heating at 90 °C for 5 min. The following gel samples were analyzed: (i) untreated gluten; (ii) gluten treated with TG; (iii) gluten–PPI blend; (iv) gluten-PP blend treated with TG. For comparison, a gel formed by heating an 18% GL-PP suspension at 100 °C for 30 min was analyzed.

#### 3.9.2. Ultrasonication Procedure

For ultrasound (US) treatment, 10 g of each protein sample suspension was placed in a plastic container (diameter 50 mm; sample layer thickness 12 mm) and sonicated in a US bath (Proclean 3.0 DSP, Ulsonix, Berlin, Germany). After treatment, the samples were cooled and stored at 4 °C for 24 h. Following storage, the texture of the gels was evaluated. The remaining portion of each sample was lyophilized and used for the qualitative and quantitative analysis of gliadin and glutenin.

### 3.10. Gel Texture Analysis

The gel texture characteristics were assessed using an acoustic device and the texture analyzer according to Zadeike et al. [53].

#### 3.10.1. The Acoustic Viscosity Meter

The acoustic viscosity meter measures the distance of sound propagation in a non-contact manner, based on the propagation time of the acoustic echolocation signal from the ultrasonic transmitter–receiver to the upper plane of the sample impact module and back. After deriving the average of the signal propagation speed values, the average distance *h* (mm) that a body falls in the analyzed gel mass per unit of time is obtained. Three measurements were performed using a metal plunger (weight 30.25 g, plate diameter—10 mm), the duration of one measurement was 300 s.

#### 3.10.2. The Texture Analyzer

The hardness (N), consistency (N·s), cohesion (N) and viscosity index (N·s) of the gels were evaluated using a texture analyzer TA.XT plus (Stable Macro Systems, Godalming, UK). A 20 mm diameter cylindrical plunger was used for the texture evaluation (penetration speed 1 mm/s, penetration depth 5 mm). Gel hardness was determined as the peak of the maximum compressive force. Viscosity index was evaluated as the area of the negative force peak. Cohesion was determined as the maximum negative force corresponding to the work required to extract the working body from the gel. Each sample was analyzed two times.

### 3.11. Gel Stability Determination

Gel stability was assessed by visually observing the retention of the ultrasonically treated sample in the test container, i.e., the degree to which the gel, after inverting the container, retains its shape for 12 s: the sample flows out within 5 s—liquid consistency; the sample bulges, falls out after 12 s—viscous consistency; the sample is stable, does not fall out after inverting after 12 s—a gel has formed. Gel elasticity was determined as the ability of the gel to return to its original shape after the deforming force has ceased to act (checked by pressing with a finger).

### 3.12. Microscopic Analysis of Gels

Microscopy of selected gel samples was performed using an electron microscope (Nikon Eclipse, Nikon Instruments Inc., Melville, NY, USA) at a magnification of 40 × 0.65.

### 3.13. Statistical Analysis

All analyses were performed at least in triplicate. The results were analyzed using Minitab version 21.4.2 software (Minitab LLC, State College, PA, USA). The analysis of variance (ANOVA) was conducted for the assessment of the suitability of the mathematical models using the coefficient of ‘*lack of fit*’ and the Fisher value (*F*). Statistical analysis of the data was performed using SPSS software (ver. 28.0, IBM, North Castle, NY, USA). The significant differences between means were evaluated by the one-way ANOVA at a significance level of 0.05.

## 4. Conclusions

Quantitative analysis of gliadin and glutenin fractions revealed that the quality of the analyzed gluten samples (expressed as GI) was significantly influenced by the gliadin fraction, as well as the ratio of high- and low-molecular-weight subunits of both gliadin and glutenin. The use of transglutaminase (TG) was proven as a more cost-effective approach and demonstrated a greater impact on increasing the wet gluten yield from wheat flour compared to the application of Shearzyme Plus hemicellulases. Both treatments effectively reduced the relative quantity of gliadins, including undesirable toxic subunits. The ability of gluten to form a strong, cohesive gel was found to be influenced by ultrasound (US) treatment duration, protein concentration in suspension, and the quality of the gluten. In all cases, higher-GI gluten samples exhibited increased firmness and consistency compared with lower-quality gluten. The firmest gels were achieved when protein concentrations were within the range of 18–20% and treated with US at 60 °C for 30 min. The addition of pea protein (PP) isolate further improved the textural properties of the gluten gels, increasing firmness and viscosity in a quality-dependent manner. Notably, the addition of PP had a more pronounced effect on the textural properties of gels made from lower-GI gluten. Moreover, with PP isolate enriched gluten gels notably increased the levels of lysine, isoleucine, and tryptophan, thereby enhancing both the nutritional quality and amino acid balance of the final product.

## Figures and Tables

**Table 1:** Gluten index (GI) values, glutenin and gliadin contents (g/100 g protein), and their quantitative ratio in commercial gluten.

Gluten Samples	GI, %	Glutenin	Gliadin	Gla/Glu
L096F	94	34.79 ± 0.76 ^c^	65.21 ± 0.45 ^b^	1.87 ^a^
F8C01	71	49.70 ± 0.84 ^d^	50.30 ± 0.60 ^a^	1.01 ^b^

Data are expressed as mean ± SD (*n* = 3). Mean values in columns with different letters are significantly different (*p* < 0.05).

**Table 2:** Glutenin and gliadin contents (g/100 g d.w.) of gluten isolated from untreated and treated-with-different-enzymes wheat flour.

Gluten Pretreatment	Time, min	Glutenin	Gliadin	Gla/Glu
Control	untreated	51.35 ± 0.17 ^cd^	48.65 ± 0.18 ^a^	0.95 ^a^
TG	30	56.35 ± 0.21 ^b^	43.65 ± 0.16 ^c^	0.77 ^c^
SH		53.75 ± 0.24 ^c^	46.25 ± 0.24 ^b^	0.86 ^b^
TG	60	61.25 ± 0.27 ^a^	38.75 ± 0.26 ^d^	0.63 ^d^
SH		52.85 ± 0.19 ^c^	47.15 ± 0.20 ^ab^	0.89 ^b^
TG	90	63.80 ± 0.21 ^a^	36.20 ± 0.23 ^e^	0.57 ^e^
SH		50.20 ± 0.18 ^d^	49.80 ± 0.17 ^a^	0.99 ^a^

Data are expressed as mean ± SD (*n* = 3). Mean values in columns with different letters are significantly different (*p* < 0.05). TG—transglutaminase, SH—Shearzyme Plus enzyme preparation.

**Table 3:** The effect of ultrasound (US) (37 kHz; 60 °C) and protein concentration on gelation capacity of different GI commercial glutens.

Protein Concentration	F8C01 (GI71)		L096F (GI94)	
	Gelation	Consistency	Gelation	Consistency
10 min US				
14%	−	liquid	−	viscous
15%	−	viscous	−	viscous
16%	−	viscous	+	gelled
18%	+	gel	+	gel
20%	+	gel	+	gel
20 min US				
14%	−	viscous	+	gelled
15%	−	viscous	+	gel
16%	+	gelled	+	gel
18%	+	gel	+	gel
20%	+	gel	+	strong gel
30 min US				
14%	−	viscous	+	gelled
15%	+	gelled	+	gel
16%	+	gel	+	gel
18%	+	strong gel	+	strong gel
20%	+	strong gel	+	strong gel

‘−’ no gel formed; ‘+’ gel formed.

**Table 4:** The amino acids (mg/g) of the gluten and gluten-PP gels pretreated by ultrasound (US) and transglutaminase (TG).

Amino Acid	GI94-PP Gels			GI71-PP Gels		
	GI94_US	US	US_TG	GI71_US	US	US_TG
Aspartic acid	12.43	13.03	14.87	11.99	12.43	13.44
Glutamine	12.74	15.47	18.45	12.82	14.21	16.82
Serine	4.12	3.93	8.08	4.49	3.17	8.18
Glycine	5.75	6.85	10.63	6.25	6.62	9.05
Alanine	3.25	4.15	6.42	2.78	4.52	5.89
Threonine	6.62	6.31	8.78	5.82	7.58	8.15
Tyrosine	9.51	9.37	10.57	8.89	9.78	10.39
Cysteine	24.68	16.62	6.66	23.78	18.18	10.1
Arginine	5.67	7.21	8.41	5.87	7.72	8.78
Tryptophan	8.77	8.89	10.97	8.57	8.79	10.43
Phenylalanine	11.06	10.92	11.51	11.50	11.22	11.36
Isoleucine	5.46	6.76	8.02	5.39	6.49	7.89
Leucine	6.56	5.72	6.68	6.53	5.37	6.47
Lysine	13.54	17.6	21.6	12.87	16.1	20.1
Methionine	4.32	5.43	9.65	4.23	5.63	10.13
Valine	11.73	18.92	21.41	10.93	17.79	22.16
Total	146.21 ^d^	157.18 ^c^	182.71 ^a^	142.71 ^e^	155.60 ^c^	179.34 ^b^

Data are expressed as means of three determinations (*n* = 3). Mean values in row with different superscript letters are significantly different (*p* < 0.05).

**Table 5:** The main quality parameters of commercial gluten samples.

Gluten Samples	Moisture, g/100 g	Protein, g/100 g	GI, %	Ash, g/100 g	WAC, g/g
L096F	6.30 ± 0.03 ^b^	77.14 ± 0.10 ^ab^	93.47 ± 0.70 ^b^	0.91 ± 0.01 ^c^	4.36 ± 0.05 ^b^
F8C01	6.92 ± 0.02 ^c^	73.43 ± 0.25 ^a^	71.08 ± 0.92 ^a^	0.86 ± 0.02 ^b^	3.92 ± 0.04 ^a^

Data are expressed as mean ± SD (*n* = 3). Mean values in columns with different superscript letters are significantly different (*p* < 0.05). GI—gluten index; WAI—water absorption capacity.

## Data Availability

Data are contained within the article or Supplementary Materials.

## Supplementary Materials

The following supporting information can be downloaded at: https://www.mdpi.com/article/10.3390/molecules30143036/s1; Figure S1: Gluten index values of gluten isolated from wheat flour after treatment with transglutaminase and hemicellulases (Shearzyme Plus); Figure S2: The electropherograms of gliadins from wheat flour treated with transglutaminase and Sherazyme (SH); Figure S3: The electropherograms of glutenins isolated from wheat flour treated with transglutaminase and hemicellulases (SH); Figure S4: Gluten and gluten–pea protein gels obtained by treating protein suspensions with ultrasound and transglutaminase; Figure S5: Prolamin profiles of untreated and treated-by-transglutaminase-and-ultrasound gluten and gluten–pea protein gels.
