# Supplementary material for: Gluten Functionality Modification: The Effect of Enzymes and Ultrasound on the Structure of the Gliadin–Glutenin Complex and Gelling Properties
Source: Molecules. 2025 Jul 19;30(14):3036. doi: 10.3390/molecules30143036 (PMC12297883; doi:10.3390/molecules30143036)
Supplement: Supplementary file 1 [file molecules-30-03036-s001.zip › molecules-3754205-supplementary.pdf]

molecules

Gluten Functionality Modification: The Effect of Enzymes and Ultrasound on the Structure of the Gliadin–Glutenin Complex and Gelling Properties

Daiva Zadeike *, Renata Zvirdauskiene and Loreta Basinskiene

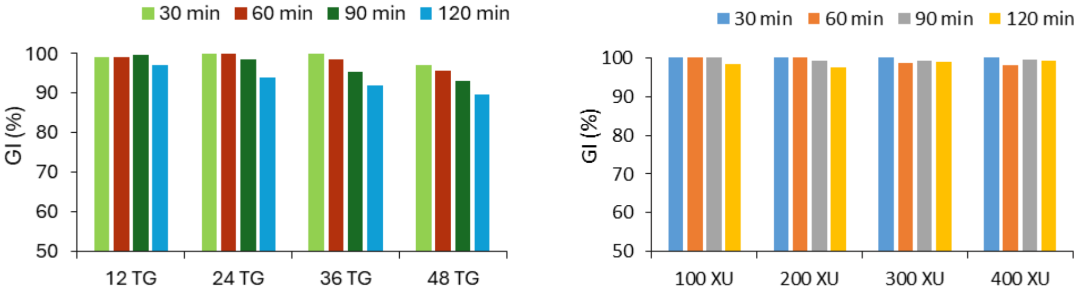

(a) (b)

Figure S1. Gluten index (GI) values of gluten isolated from wheat flour after treatment with transglutaminase (TG) (a) and hemicellulases (Shearzyme Plus) (b)

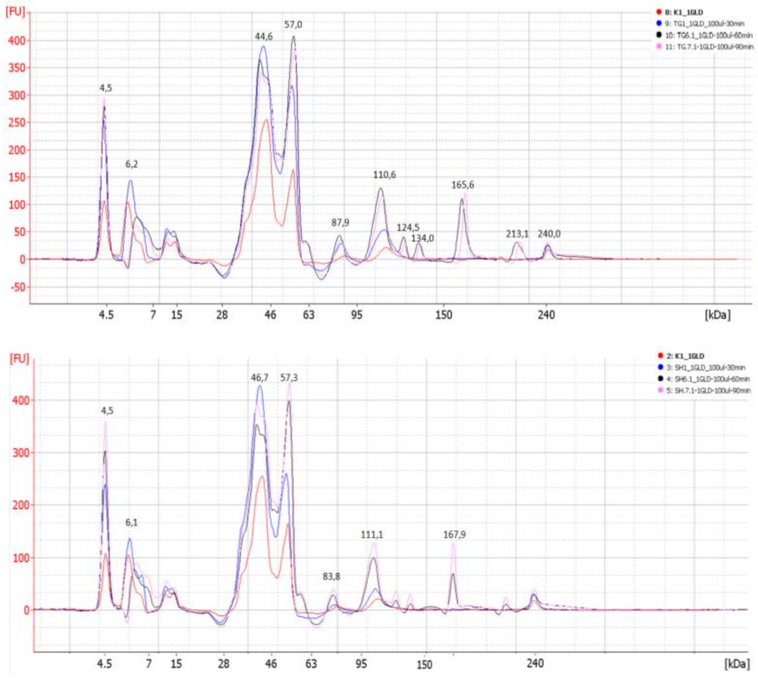

(a) (b)

Figure S2. The electropherograms of gliadins from wheat flour treated with transglutaminase (TG) (a) and Sherazyme (SH) (b). The red line represents the untreated wheat gluten (K).

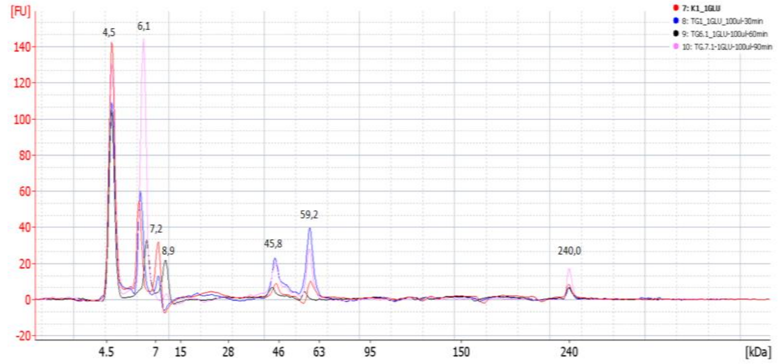

(a)

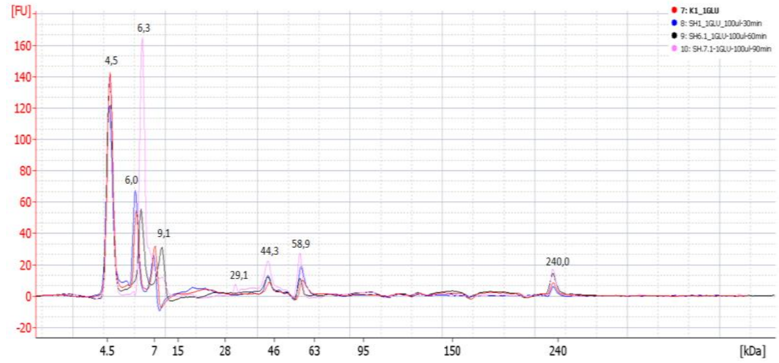

(b)

Figure S3. The electropherograms of glutenins isolated from wheat flour treated with transglutaminase (TG) (a) and hemicellulases (SH) (b). The red line represents the untreated wheat gluten (K).

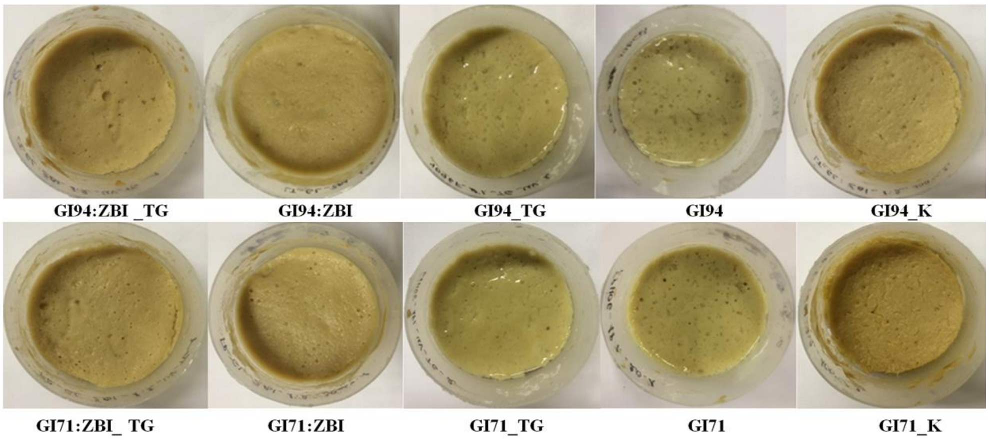

Figure S4. Gluten and gluten-pea protein (ZBI) gels obtained by treating protein suspensions with ultrasound (US) and transglutaminase (TG). K – gluten-pea protein (ZBI) (1:2 ratio) gel obtained by heating the protein suspension at 100 °C.

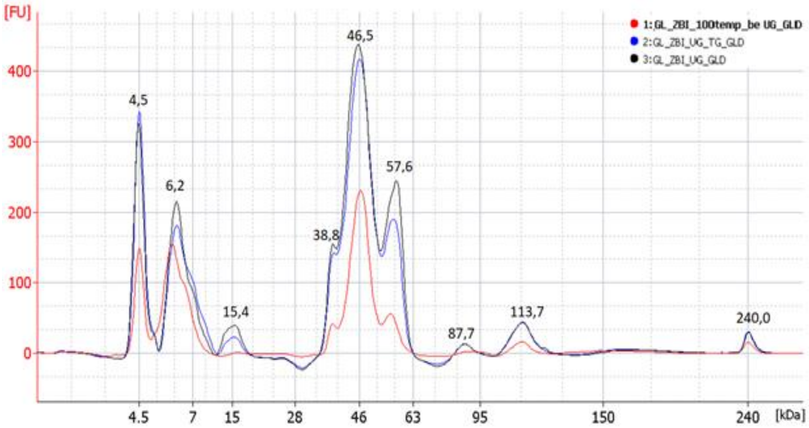

(a)

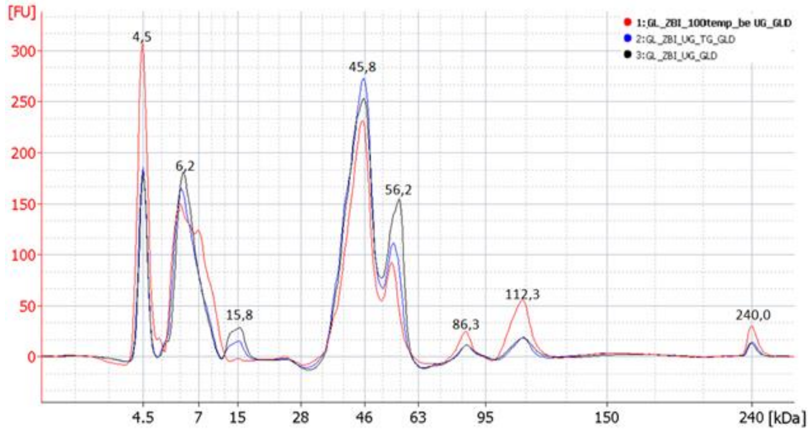

(b)

Figure S5. Prolamin profiles of untreated and treated by transglutaminase (TG) and ultrasound (US) gluten GI94 (a) and GI71(b), and gluten-pea protein (ZBI) (1:2) gels. The red line represents gluten-pea protein gel, obtained by heating protein suspension at 100 ^{o}C.
